# Anesthetic management using high-flow nasal cannula therapy during cardiac catheter examination of a neonate with hypoplastic left heart syndrome

**DOI:** 10.1186/s40981-022-00572-x

**Published:** 2022-10-12

**Authors:** Yoshiaki Ito, Tomonori Yamashita, Kazuya Tachibana

**Affiliations:** 1 Department of Anesthesiology, National Cerebral and Cardiovascular Center, 6-1 Kishibe-Shimmachi, Suita, Osaka 564-8565 Japan; 2 Department of Anesthesiology, Osaka Women’s and Children’s Hospital, Izumi, Osaka Japan

**Keywords:** High-flow nasal cannula therapy, Pediatric anesthesia, Hypoplastic left heart syndrome, Congenital heart disease, Monitored anesthesia care

## Abstract

**Background:**

Sedation during cardiac catheter examination in neonates with complex congenital heart disease is challenging, as even the slightest change in the circulatory or respiratory status can lead to hemodynamic collapse. Here, we report a case wherein we achieved adequate sedation with a high-flow nasal cannula (HFNC) for catheter examination in a neonate with a congenital cardiac anomaly.

**Case presentation:**

An 11-day-old boy with hypoplastic left heart syndrome was scheduled for a cardiac catheter examination prior to the Norwood procedure. He underwent bilateral pulmonary artery banding (PAB) on day 1 and was receiving dobutamine, milrinone, alprostadil, and dexmedetomidine in addition to air and nitrogen insufflation via HFNC, which was applied following extubation on day 3 and nitrogen therapy on day 6 owing to persistent pulmonary overcirculation symptoms (tachypnea and low arterial blood pressure) despite bilateral PAB. A catheter examination was performed on day 11 with careful monitoring of expired carbon dioxide and observation of chest wall motion. Adequate sedation was provided with supplemental midazolam and fentanyl along with HFNC without tracheal intubation.

**Conclusions:**

The findings from this case suggest that HFNC is a safe and effective tool for oxygenation during cardiac catheter examination under sedation in neonates.

## Background

The effectiveness of high-flow nasal cannula (HFNC) therapy for post-extubation respiratory support in neonates is well known [1, 2]. However, there are few reports of HFNC use in the cardiac catheter examination of neonates with complex congenital heart disease. Hypoplastic left heart syndrome (HLHS) is one of the most severe congenital heart diseases [3, 4]. Patients with HLHS require strict control of various hemodynamic factors, including arterial pH, partial pressure of oxygen and carbon dioxide, and respiratory conditions such as airway pressure and respiratory system compliance that have an impact on pulmonary vascular resistance (PVR).

Here, we successfully managed anesthesia for the cardiac catheter examination of a newborn patient with HLHS without tracheal intubation using HFNC and careful monitoring. Written informed consent for this publication was obtained from the patient’s family.

## Case presentation

We report the case of an 11-day-old boy (height, 49 cm; weight, 3.65 kg) with a prenatal diagnosis of HLHS. He was born via spontaneous vaginal delivery (gestational age, 40 weeks; birth weight, 4.00 kg; Apgar scores, 8/8 [1 min/5 min]) and diagnosed with HLHS with mitral atresia and aortic stenosis without abnormalities involving organs other than the heart.

In the pediatric intensive care unit (PICU), he received continuous alprostadil and dobutamine infusion to maintain systemic blood flow using a peripherally inserted central catheter (PICC) secured in the right upper limb. Balloon atrial septostomy was not performed because there were no signs of a restrictive patent foramen ovale. On day 1, bilateral pulmonary artery banding (PAB) was performed. HFNC therapy (Optiflow Junior 2^TM^, prong size M, Fisher and Paykel Healthcare, Auckland, New Zealand) was applied following extubation on day 3 and nitrogen (N_2_) therapy on day 6 owing to persistent pulmonary overcirculation symptoms (tachypnea and low arterial blood pressure) despite bilateral PAB.

Cardiac catheterization was scheduled for day 11 of life, for preoperative evaluation for a Norwood procedure. Before this procedure, he received dobutamine 6.7 μg/kg/min, milrinone 0.73 μg/kg/min, alprostadil 5 ng/kg/min, and dexmedetomidine 1.25 μg/kg/h through the PICC. Vital signs were as follows: transcutaneous oxygen saturation (SpO_2_), 80–85% (HFNC air 10 L/min + N_2_ 1 L/min, F_I_O_2_: 0.19; no difference between the upper and lower limbs); respiratory rate, 80–100 breaths/min; heart rate, 145 beats/min (sinus rhythm); and blood pressure, 77/40 mmHg. Arterial blood gas analysis showed a pH of 7.429, PaCO_2_ of 43.9 mmHg, base excess of 4.0, and PaO_2_ of 39.7 mmHg. The cardiothoracic ratio on chest radiography was 55%, and tricuspid regurgitation was trivial on echocardiography.

Considering the risk of cardiovascular collapse (e.g., hypotension due to pulmonary overcirculation) associated with general anesthesia and positive pressure ventilation and potential complications due to prolonged mechanical ventilation, after consultation with the surgeon, we decided that HFNC would have significant advantages from scientific and clinical perspectives and selected this modality for our patient.

### Anesthetic process

For the management of anesthesia without intubation using HFNC, the following two strategies were performed to secure stable spontaneous ventilation: (1) the patient’s head was extended, with a shoulder roll used to maintain the upper airway and (2) the patient underwent capnography through a sampling line connected to an 8-Fr suction tube tip inserted in the pharynx to detect any airway obstruction or respiratory depression during the procedure.

**Fig. 1:**
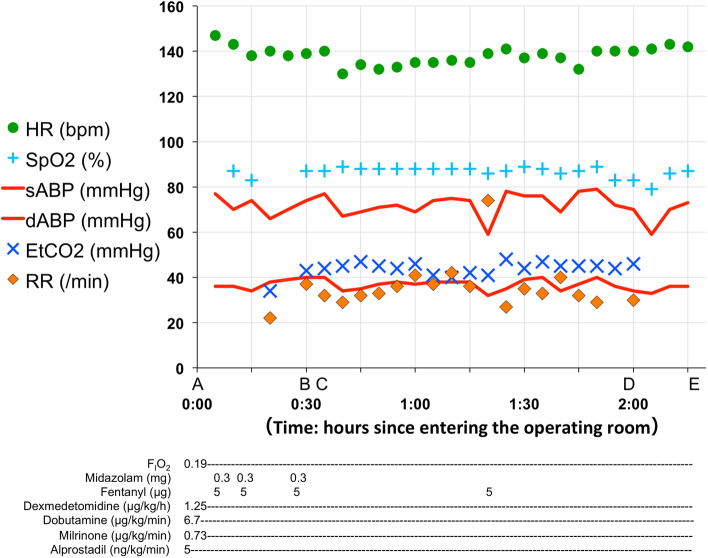
Hemodynamic changes in the patient during cardiac catheterization upon **A** entry into the operating room, **B** initiation of examination/vascular puncture, **C** insertion of the 5-Fr sheath/arterial blood gas analysis, **D** completion of the examination, and **E** exit from the operating room. sABP, systolic arterial blood pressure; dABP, diastolic arterial blood pressure

Midazolam (total 0.27 mg/kg) and fentanyl (total 4.1 μg/kg) were titrated to safely increase the sedation level while considering the respiratory rate, chest wall movement, and SpO_2_ level. Following sufficient sedation and stable spontaneous ventilation (respiratory rate 30–40 breaths/min), the surgeon anesthetized the insertion site with 1% lidocaine. A 5-Fr sheath was placed in the right femoral vein, followed by an examination. The arterial blood gas analysis at treatment initiation revealed a pH of 7.378, PaCO_2_ of 44.9 mmHg (EtCO_2_, 42 mmHg), base excess of 0.9, and PaO_2_ of 48.3 mmHg. Cardiac catheter examination showed the following: Qp/Qs, 1.72; Rp, 2.39 Wood units・m^2^; and Rp/Rs, 0.14. No body movement was observed during the examination. Cardiac catheterization was completed without upper airway obstruction, apnea, or substantial oxygen desaturation (Fig. 1). Approximately 5 h after the examination, the respiratory rate returned to 80–100 breaths/min. Respiratory support with HFNC therapy was continued in PICU after the catheter examination until the day of the Norwood procedure.

## Discussion

Neonates with complex cardiac malformations have poor tolerance for cardiac preload and afterload; therefore, slight deviations in systemic vascular resistance, PVR, and fluid balance from the normal safe range can lead to fatal cardiac collapse.

HFNC use during cardiac catheterization under spontaneous breathing in neonates with HLHS prior to surgical repair is advantageous in the following manner. First, HFNC provides a fresh gas flow rate higher than that of patients’ inspiration and therefore secures the constant actual F_I_O_2_ regardless of patients’ breathing pattern [5], which must remain constant during cardiac catheterization because PVR may be affected. Second, HFNC reduces the anatomical dead space through the washout effect in the upper airway [6] and is expected to partially offset the impact of anesthesia on carbon dioxide retention, which may affect PVR and patients’ breathing [7–9]. Third, the positive pressure generated by HFNC can maintain the patency of the upper airway and avoid the large intrathoracic negative pressure and increased venous return blood volume caused by strong respiratory effort against the increased airway resistance under sedation. Fourth, HFNC has the effect of continuous positive airway pressure and can be expected to maintain peripheral airway patency under conditions of weak spontaneous breathing during procedures, which can prevent alveolar collapse [2, 10] and increase PVR. In the present case, the patient had pulmonary overcirculation before the examination and decreased pulmonary compliance due to alveolar edema and atelectasis. Under these conditions and without HFNC, induction of deep sedation may result in decreased ventilation, additional atelectasis, carbon dioxide retention, and acidemia, eventually leading to increased PVR. We used HFNC with the aim of maintaining the pre-examination PVR throughout the examination. Although the respiratory rate decreased from 80 to 30 breaths/min due to sedation during the examination, we were able to maintain oxygenation and PaCO_2_ by using HFNC. The lack of significant differences in vital signs or blood gas analysis before and during the examination indicated that we successfully minimized changes in PVR. Therefore, HFNC therapy allows even neonates with pulmonary overcirculation to maintain stable spontaneous breathing and hemodynamics during a sedated examination. General anesthesia with intubation and mechanical ventilation was avoided because of various reasons. First was the risk of decreased PVR due to inappropriate manual ventilation during induction of anesthesia, which could cause further pulmonary overcirculation and result in hypotension and shock. The second was the risk of decreased cardiac output due to decreased preload caused by positive pressure ventilation. Third, the risk of myocardial ischemia due to reduced coronary artery blood flow is high in patients with HLHS. The fourth was the risk of pneumonia if weaning from mechanical ventilation became difficult.

Despite the difficulty in performing capnography in non-intubated patients, the insertion of a sampling line in the pharynx can detect airway obstruction and apnea. We found little disparity between EtCO_2_ and PaCO_2_ in this case, indicating that a sampling line in the patient’s pharynx may also be used to predict poor minute ventilation in patients with spontaneous ventilation. The patient required strict respiratory control, so we used capnography as a general monitoring tool for respiratory depression and arrest [11]. We also measured PaCO_2_ using blood gas analysis as needed.

The key point in the successful management of this case was the safe induction of anesthesia by administering anesthetic agents while carefully observing chest wall movement and respiratory rate to confirm effective spontaneous respiration. The choice and dosage of sedatives and analgesics require careful consideration when performing an examination or treatment with spontaneous breathing. Our patient received continuous high-dose dexmedetomidine in PICU. We administered dexmedetomidine throughout the examination because it is considered safe for use as a sedative without any hemodynamic or respiratory effects during cardiac catheterization [12]. Furthermore, dexmedetomidine reportedly does not affect PVR when used to sedate children with pulmonary hypertension [13]. We considered that sedation with dexmedetomidine alone would be inadequate; therefore, we added midazolam and fentanyl (familiar agents for us), because they are less associated with cardiac depression and can be antagonized with reversal agents in case of unexpected apnea.

To summarize, we successfully managed a neonate with a complex cardiac malformation under intravenous anesthesia using HFNC therapy during a non-intubated cardiac catheter examination.

## Data Availability

Not applicable.

## Abbreviations

HFNC: High-flow nasal cannula
HLHS: Hypoplastic left heart syndrome
PVR: Pulmonary vascular resistance
PICU: Pediatric intensive care unit
PICC: Peripherally inserted central catheter
PAB: Pulmonary artery banding
